# Recent Advances in Nickel Catalysts for Sonogashira Coupling

**DOI:** 10.1002/open.70217

**Published:** 2026-05-10

**Authors:** Sara Payamifar, Pegah Etminan, Ahmad Poursattar Marjani

**Affiliations:** ^1^ Department of Organic Chemistry Faculty of Chemistry Urmia University Urmia Iran; ^2^ Department of Chemistry Faculty of Science Atatürk University Erzurum Turkey

**Keywords:** aryl halides, coupling reaction, nickel catalyst, sonogashira reaction, terminal alkynes

## Abstract

The Sonogashira coupling, catalyzed by transition metals, has become a valuable method for forming carbon‐carbon bonds. While palladium‐copper was originally recognized as the most effective catalyst for this transformation, recent advances have highlighted the growing prominence of nickel‐catalyzed Sonogashira reactions. Nickel has gained considerable interest due to its lower cost and more environmentally friendly profile compared to palladium. This review focuses on recent progress in nickel‐catalyzed Sonogashira coupling, surveying studies published between 2021 and 2025.

## Graphical abstract

The Sonogashira coupling, catalyzed by transition metals, has become a valuable method for forming carbon‐carbon bonds. Nickel has gained considerable interest due to its lower cost and more environmentally friendly profile compared to palladium. This review focuses on recent progress in nickel‐catalyzed Sonogashira coupling, surveying studies published between 2021 and 2025.

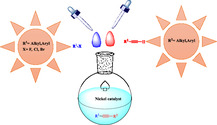

**Abbreviations**
Ar—X: Aryl halide
C—C: Carbon—carbon
Nitmtaa: Dibenzotetramethyltetraazaannulene
DFNS: Dendritic fibrous nanosilica
CPTES: 3‐Chloropropyltriethoxysilane
C_11/_H_12_N_4_
: 2,4‐Diamino
PVPy: Polyvinylpyridine
DMC: Dimethyl carbonate
HTE: High‐throughput experimentation
DoE: Design‐of‐experiments
TOF: Turnover frequency
TON: Turnover number
XPS: X‐ray photoelectron spectroscopy
EDX: Energy‐dispersive X‐ray
XRD: X‐ray diffraction
FT‐IR: Fourier transform infrared spectroscopy
SEM: Scanning electron microscopy
TEM: Transmission electron microscopy
TGA: Thermal gravimetric analysis
BET: Brunauer‐Emmett‐Teller
VSM: Vibrating‐sample magnetometry
ICP: Inductively coupled plasma

## 1 Introduction

Green chemistry drives both academic and industrial research, emphasizing the demand for safer chemicals alongside eco‐friendly, affordable, and biodegradable materials. Catalysts play a pivotal role in diverse organic transformations, amplifying their relevance in sustainable practices. Thus, developing clean, environmentally benign synthetic protocols with green solvents—avoiding toxic organic alternatives—remains essential [1, 2, 3].

Coupling reactions involve two distinct components reacting in the existence of a catalyst to form a new bond, representing an extremely valuable class of transformations in organic synthesis. These reactions provide a powerful tool for efficient organic synthesis, enabling the assembly of complex molecules from simple starting materials. As a result, they find widespread industrial application, particularly in chemical manufacturing. Coupling reactions are typically divided into two primary categories: cross‐coupling, which links two different substrates, and homocoupling, involving two identical substrates. These reactions employ various catalysts, among which Pd‐based systems exhibit the broadest applicability [4, 5]. Transition metals hold a central role in organic synthesis as essential catalysts for many transformations. Pd stands out for its prowess in facilitating the construction of carbon‐carbon (C–C) and carbon‐heteroatom bonds. Reactions such as Suzuki, Sonogashira, and Heck catalyzed by Pd have emerged as cornerstones, highly efficient strategies for C—C bond assembly. Recently, both homogeneous and heterogeneous Pd catalysts have been widely used for these reactions [6, 7, 8, 9, 10].

**SCHEME 1:**
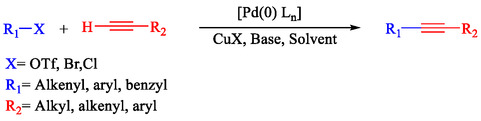
The Sonogashira coupling.

The Sonogashira coupling is undeniably one of the most useful and widely utilized procedures in organic chemistry. This well‐known reaction has found broad applications across various fields, such as the construction of heterocyclic compounds, natural products, pharmaceuticals, bioactive molecules, nanomaterials, and numerous other materials integral to everyday life [11, 12, 13]. The Sonogashira reaction is one of the leading techniques for forming sp^2^‐carbon to sp‐carbon bonds and is broadly applied in organic synthesis. Traditionally, this reaction involves coupling a terminal alkyne with an alkyl halide using a palladium catalyst, a copper complex (cocatalyst), and a phosphine or amine (base). This distinctive combination of reagents allows the reaction to proceed efficiently at moderate conditions (Scheme 1) [14, 15, 16].

**SCHEME 2:**
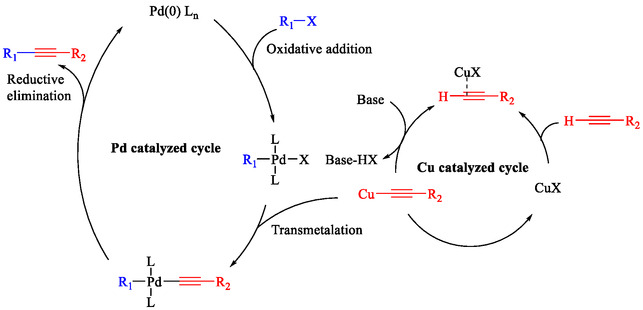
The way presented for the Sonogashira reaction.

The Sonogashira coupling is a highly valuable technique for synthesizing functionalized alkynes. Since its initial report in 1975, which employed the PdCl_2_(PPh_3_)_2_ catalyst in combination with a CuI cocatalyst, significant progress has been made in palladium‐catalyzed Sonogashira reactions, greatly expanding both its versatility and its range of applications [17, 18, 19, 20, 21]. A key limitation of palladium catalysis is the high cost of noble Pd catalysts and the challenging recovery process. Additionally, potential palladium contamination in the final products restricts its application in bioactive compounds and raises environmental and economic issues, especially in large‐scale industrial syntheses [22, 23, 24]. A possible pathway for the Sonogashira coupling has been presented. As shown in Scheme 2, this reaction likely flows *via* interconnected Pd‐ and Cu‐catalyzed cycles.

**SCHEME 3:**
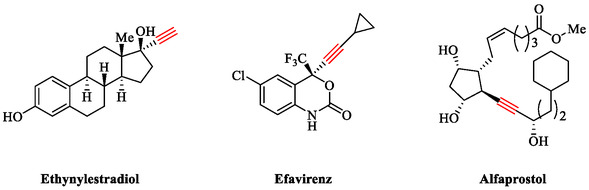
The drugs featuring alkynyl groups.

Alkynes are extremely valuable functional groups in organic synthesis, serving as versatile synthetic intermediates for diverse transformations and as key structural elements in numerous natural products, bioactive compounds, and organic materials. Incorporating an alkyne moiety into drug molecules can significantly improve biological properties, including bioavailability, metabolic stability, and lipophilicity (Scheme 3). Aryl‐alkynes and conjugated enynes, key products of the Sonogashira coupling, are widely used in natural product synthesis, pharmaceuticals, polymers, and heterocycle chemistry. Conventionally, this coupling relies on palladium catalysts paired with copper cocatalysts, often in combination with various ligands and additives, under homogeneous conditions to boost product yields. Copper cocatalysts and phosphine ligands typically require high temperatures, and while copper enhances reactivity in oxygen‐free environments, it also generates side products, such as bi‐alkynes, *via* the Glaser reaction [25, 26, 27, 28, 29, 30, 31, 32, 33, 34].

Various transition metal complexes, including those of Cu [35, 36], Co [37, 38], Fe [39], Au [40], and Ru [41], have been used in the Sonogashira reaction. In modern synthetic chemistry, transition‐metal catalysis, particularly with nickel, plays an important role. Nickel, an abundant, cost‐effective metal with high catalytic potential, plays a key part in organic synthesis, especially in diverse coupling reactions. Traditionally, nearly all such reactions have relied on Pd catalysts. Although these reactions have traditionally employed Pd catalysts, Pd‐based procedures suffer from several drawbacks: high cost, significant risk of catalyst contamination at the end of the catalytic cycle, and palladium's toxicity, which limits its use in the synthesis of bioactive compounds. These limitations impose significant economic and environmental hurdles for Pd‐catalyzed coupling reactions. Consequently, from a green chemistry perspective, contemporary synthetic chemistry advocates for less toxic, recyclable, Pd‐free catalytic systems, a pressing challenge for modern organic chemists [42, 43].

Ni, which belongs to the same group as palladium, offers a less toxic and more cost‐effective alternative catalyst. Nickel, which is in the same group as palladium, provides a less harmful and more affordable alternative catalyst. It boasts high catalytic activity and the ability to access various oxidation states. Furthermore, nickel's greater electropositivity facilitates easier oxidative addition while hindering β‐hydride elimination, thanks to a higher rotational energy barrier in Ni—C bonds relative to Pd [44]. These attributes position Ni as an appealing Pd substitute.

Over the past two decades, nickel complexes have been extensively investigated as catalysts for C–C coupling reactions, including the Suzuki coupling [45, 46], Kumada coupling [47, 48, 49, 50], Negishi reaction [51], amination reactions [52], and Heck reaction [53].

Beletskaya et al. first reported Ni complexes for the Sonogashira coupling reaction. Since that time, various Ni complexes have been extensively studied for their catalytic performance, making this field a vibrant area of research [51]. Key applications include C(sp)—C(sp^2^) and C(sp)—C(sp^3^) bond formations, carbonylative Sonogashira reactions, and the preparation of biologically active compounds [11, 54, 55].

## 2 Application of Ni‐Catalyzed for Sonogashira Reactions

Ni‐catalyzed Sonogashira coupling couples terminal alkynes and aryl or alkyl halides, forming C(sp)—C(sp^2^) or C(sp)—C(sp^3^) bonds under milder, more sustainable conditions than traditional Pd catalysts. Nickel serves as an earth‐abundant, less toxic alternative, enabling high activity in both homogeneous and heterogeneous systems. Nair et al. have published a review of Ni‐catalyzed Sonogashira coupling. This study presents the latest methods and the potential of nickel‐catalyzed Sonogashira reactions in both homogeneous and heterogeneous systems, and it encompasses the literature up to 2020 [56]. In this regard, we examine Ni‐catalyzed Sonogashira reactions, covering the literature from 2021 up to 2025.

**SCHEME 4:**
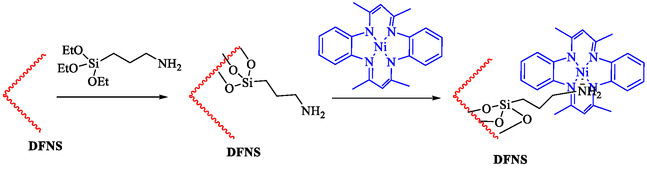
The method of synthesis of Nitmtaa@DFNS nanocatalyst.

Tian et al. introduced a reusable nanocatalyst, Nitmtaa@DFNS, comprising nickel(II) dibenzotetramethyltetraazaannulene (Nitmtaa) immobilized on amino‐functionalized dendritic fibrous nanosilica (DFNS) via Ni—N bonds for the carbonylative Sonogashira reaction of aryl iodides with terminal alkynes under carbon monoxide (2 MPa) [57]. The procedure for preparing the Nitmtaa@DFNS nanocatalyst is shown in Scheme 4. The catalyst enables good‐to‐excellent yields (76%–96%) of alkynones in DMF with K_3_PO_4_ as the base at reflux for 4 h, using just 6 mg of catalyst loading (Table 1). DFNS nanoparticles (250–300 nm diameter, fibrous morphology, 634 m^2^/g BET area) were synthesized hydrothermally, aminated with 3‐aminopropyltriethoxysilane, and loaded with Nitmtaa. Post‐immobilization properties include reduced surface area (299 m^2^/g), confirmed pores (8 nm average), and elements (Si, O, C, N, Ni) *via* transmission electron microscopy (TEM)/scanning electron microscopy (SEM), Fourier transform infrared spectroscopy (FT‐IR) (C–N/C–C at 1615/1458 cm^−1^), X‐ray photoelectron spectroscopy (XPS), energy‐dispersive X‐ray (EDX), X‐ray diffraction (XRD) (amorphous silica), thermal gravimetric analysis (TGA) (21% organic loss), and vibrating‐sample magnetometry (VSM) (magnetic recoverability). Optimal conditions outperformed polar protic solvents, other bases, or solvent‐free setups; yields peaked at 93% for iodobenzene/phenylacetylene. Scope tolerated electron‐withdrawing (CF_3_, CN; 76%–80%), donating (Me; 91%–95%), and halo (Cl, Br, I) substituents on aryl iodides. Control tests confirmed Nitmtaa's role over supports alone. The catalyst recycled ten times with minimal yield drop (93 to 89%), retaining fibrous structure (SEM/TEM) and thermal stability (TGA). Hot filtration and leaching tests verified true heterogeneity, with no activity post‐removal.

**TABLE 1:** Sonogashira reactions using Nitmtaa@DFNS.

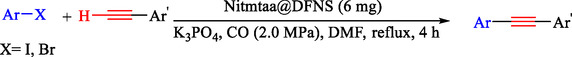			
Entry	Ar‐X	Product	Yield, %
1	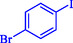	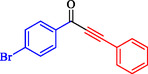	83
2	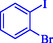	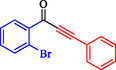	79
3	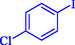	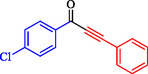	86
4	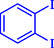	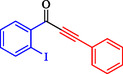	81
5	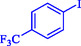	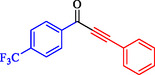	76
6	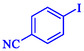	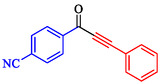	80
7	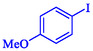	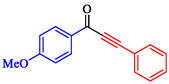	96
8	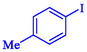	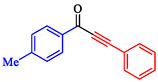	95
9	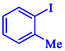	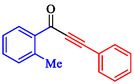	91

*Note:* Reaction condition: Ar–X (1.0 mmol), terminal alkyne (1.2 mmol), catalyst (6 mg), K_3_PO_4_, CO (2.0 MPa), and DMF, reflux, 4 h.

**SCHEME 5:**
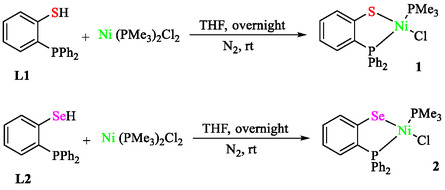
The preparation of nickel chlorides **1** and **2**.

**SCHEME 6:**
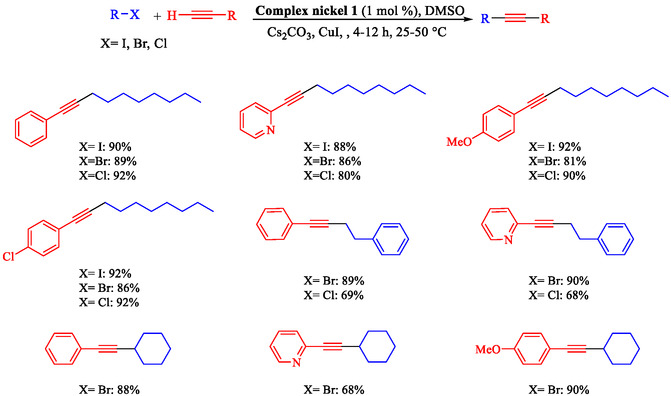
Sonogashira reactions of alkynes and alkyl halides.

Fan et al. reported nickel‐catalyzed Sonogashira Csp^3^–Csp coupling reactions between nonactivated alkyl halides (iodides, bromides, chlorides) and terminal alkynes, using P–, S–, and P, Se‐chelated Ni(II) chloride complexes under mild conditions [58]. The preparation of nickel chlorides 1 and 2 is exhibited in Scheme 5. Complex 1 (P, S‐chelate) outperforms complex 2, enabling low catalyst loadings (1 mol%) with CuI cocatalyst, Cs_2_CO_3_ base, and DMSO solvent (Scheme 6). Complexes 1 and 2 form from NiCl_2_(PMe_3_)_2_ and bidentate ligands (2‐diphenylphosphanylbenzenethiol or selenol) in THF, yielding air‐stable square‐planar structures confirmed by X‐ray crystallography (Ni–Cl ∼ 2.20 Å, trans P atoms). Optimization with 1‐iodooctane and phenylacetylene achieved 92% yield at 25°C in 4 h, bromides at 40°C (8 h), and chlorides at 50°C (12 h) with NaI additive. Aryl/heteroaryl alkynes coupled with primary/secondary/tertiary alkyl iodides (88%–92% yields), bromides (68%–94%), and chlorides (49%–97%), tolerating OMe, Cl, OH, NH_2_, and esters. Aliphatic alkynes gave moderate yields (19%–47%). Gram‐scale chlorooctane coupling succeeded (91% yield). Advantages and context This system advances prior Ni catalysts by activating alkyl chlorides at 50°C (vs. 100°C–140°C), using simple chelate ligands rather than pincer ligands, and exhibiting broad functional‐group tolerance for alkyne synthesis in materials/drugs.

**SCHEME 7:**
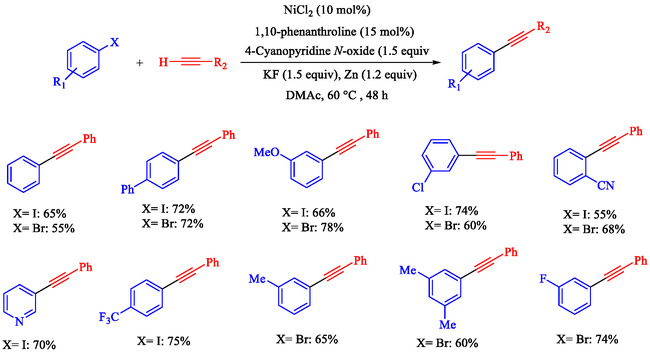
Ni‐catalyzed Sonogashira reaction.

Chen et al. reported a nickel‐catalyzed enantioselective Heck‐Sonogashira annulation/alkynylation for forming Csp^3^—Csp bonds and a related Sonogashira coupling for Csp^2^—Csp bonds, using a single Ni catalyst without copper cocatalysts [59]. The methods target 3,3‐disubstituted‐2‐oxoindoles and other alkyne‐containing structures relevant to natural products and pharmaceuticals. The authors optimized chiral phosphine‐oxazoline ligands for the tandem reaction, achieving up to 93% ee with NiCl_2_, a Zn reductant, and 4‐cyanopyridine *N*‐oxide as the base in NMP at 80°C. For Sonogashira coupling, 1,10‐phenanthroline enabled a broad scope of aryl iodides and bromides at 60°C–70°C in DMAc (Scheme 7). These overcome Ni's strong alkyne coordination, previously requiring noble metals or cocatalysts. Annulationalkynylation tolerated diverse arylacetylenes, iodoacrylamides (including α‐substituted for quaternary centers), and heteroaryls, yielding products in 50%–88% with 86%–95% ee. Sonogashira variants tolerated electron‐rich/poor aryl halides (Ar–X) and protected alkynes, affording 40%–90% yields. Products underwent deprotection, click chemistry, cyanation, and hydrogenation, demonstrating utility for oxindole synthesis. Density functional theory (DFT) calculations identified the Csp^3^–Csp coupling as rate‐limiting (26.1 Kcal/mol barrier), with amide oxygen aiding alkyne proton abstraction. A di‐phosphine‐chelated β‐alkyl‐Ni^II^–I resting state was isolated and crystallized, confirming prealkyne complexation. Sonogashira DFT showed Ni‐activated alkyne deprotonation (16.0 Kcal/mol barrier).

Keskin et al*.* explained that PdNiFe_2_O_4_, consisting of Pd nanoparticles (2.0 wt%) supported on magnetic nickel ferrite, serves as a highly effective, reusable catalyst in Sonogashira reactions between terminal alkynes and aryl iodides [60]. This catalyst outperforms reported Pd‐based magnetic systems, achieving a turnover frequency of 106.4 h^−1^ for phenylacetylene and iodobenzene in ethanol at 70°C and achieving near‐complete conversion in 1 h without copper cocatalysts. PdNiFe_2_O_4_ is prepared via a simple impregnation‐reduction method: NiFe_2_O_4_ powder is impregnated with aqueous Pd(NO_3_)_2_, followed by NaBH_4_ reduction at room temperature. Characterization by TEM, FE‐SEM, XRD, XPS, and ICP‐OES confirms the uniform dispersion of Pd nanoparticles (3–5 nm) on the spinel NiFe_2_O_4_ support, with Pd^0^ species and increased oxygen defects enhancing electron transfer and stability. The catalyst couples aryl iodides and phenylacetylene or 1‐hexyne, utilizing K_2_CO_3_ as base in ethanol or THF at 70°C, yielding products such as 1,2‐diphenylethyne (71–97% isolated) and alkyl‐aryl derivatives (63%–85%, Table 2). Electron‐withdrawing groups on aryl iodides accelerate reactions; activity persists in water (85% GC yield after 24 h). PdNiFe_2_O_4_ can be recycled up to ten times with minimal Pd leaching, retaining 95% conversion *via* magnetic separation. The mechanism involves oxidative addition of an aryl iodide, alkyne deprotonation/base‐assisted coordination, transmetalation, and reductive elimination, facilitated by the redox properties of NiFe_2_O_4_. Table 3 shows a comparison of the Pd/NiFe_2_O_4_ catalyst with other catalysts formerly reported for the Sonogashira coupling.

**TABLE 2:** The catalytic performance of the Pd/NiFe_2_O_4_ catalyst in Sonogashira reactions.

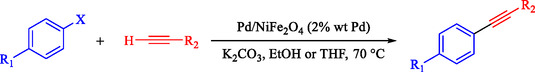						
Entry	Acetylene	**Ar**–**X**	Product	Solvent	Time, h	Yield, %
1	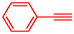	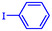	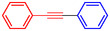	EtOH	1	71
2	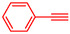	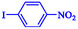	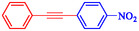	THF	2.5	97
3	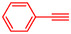	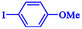	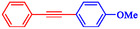	EtOH	3	80
4	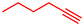	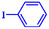	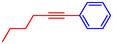	EtOH	1	63
5	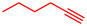	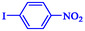	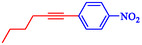	THF	2.5	76
6	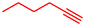	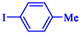	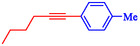	EtOH	2	80
7	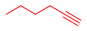	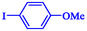	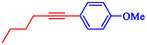	EtOH	24	81
8	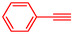	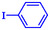	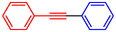	H_2_O	24	85

*Note:* Reaction condition: Ar–X (1 mmol), acetylene (1.05 mmol), K_2_CO_3_ (2 mmol), Pd/NiFe_2_O_4_ (2% wt Pd) (50.0 mg), EtOH or THF (3 mL), 70°C.

**TABLE 3:** A comparison of the Pd/NiFe_2_O_4_ catalyst and earlier catalysts used in the Sonogashira coupling between phenylacetylene and iodobenzene.

Entry	Catalyst	Solvent	T, °C	Base	Time, h	**TOF, h** ^**−1**^	Yield, %	Ref.
1	Pd_3_Cu_1_/SiC	DMF	60	Cs_2_CO_3_	8	—	2	[61]
2	Pd/Fe_3_O_4_@GON	DMSO	120	NaOAc	0.5	62.1^a^	99	[62]
3	Ni/rGO	NMP/CuI	120	K_2_CO_3_	4	—	95	[63]
4	Co‐MS@MNPs/CS	DMSO	140	KOH	10	—	72	[64]
5	Pd‐MNPSS	H_2_O	100	K_2_CO_3_	10	—	87	[65]
6	Pd‐Co/Graphene	THF/H_2_O/CuI	80	Et_3_N, PPh_3_	12	4.1^a^	98	[66]
7	Pd‐phosphinite polymer (0.16)	H_2_O	60	DABCO	24	—	89	[67]
8	Pd_3_Cu1/SiC under Xe lamp	DMF	60	Cs_2_CO_3_	8	8.9^a^	99	[61]
**9**	**Pd/NiFe_2_O_4_**	**EtOH**	**70**	**K_2_CO_3_**	1	**106.4**	**99**	**[** **60** **]**
**10**	**Pd/NiFe_2_O_4_**	**H_2_O**	**70**	**K_2_CO_3_**	**24**	**—**	**85**	**[** **60** **]**

a TOF = mmol product × (mmol Pd)^‐1^ × (time)^−1^

**SCHEME 8:**
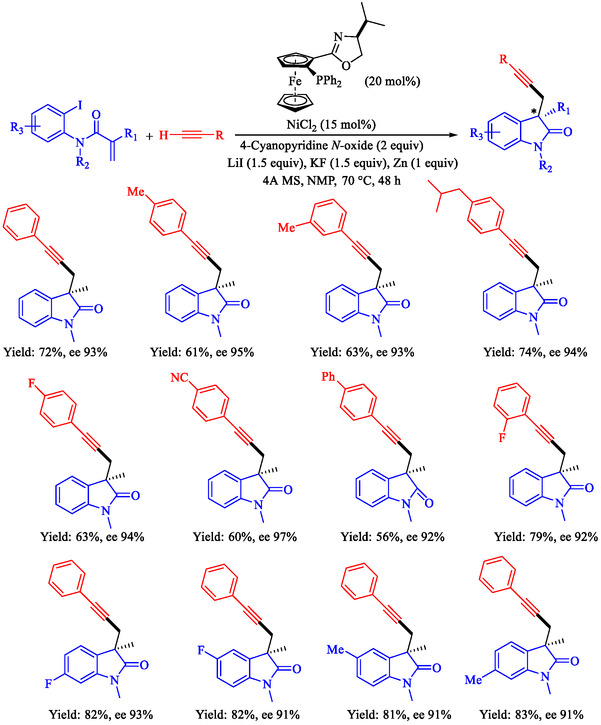
Ni‐catalyzed enantioselective domino Heck/Sonogashira coupling.

Chen et al*.* reported a copper‐free nickel‐catalyzed Sonogashira reaction that uses deaminative activation of alkylpyridinium salts (Katritzky salts derived from primary, secondary, and benzyl amines) and terminal alkynes to form C(sp^3^)—C(sp) bonds and facilitate the preparation of alkyl‐substituted alkynes [68]. Researchers developed an air‐stable amide‐type NN^2^ pincer ligand (L4: 6‐methyl‐*N*‐(quinolin‐8‐yl) picolinamide) that stabilizes NiCl_2_ · 6H_2_O precatalyst, achieving > 90% yields at 80°C in THF with K_3_PO_4_ (Scheme 8). This overcomes challenges such as β–H elimination and alkyne homocoupling, unlike prior Pd/Ni systems, which were limited to alkyl halides. The method tolerates diverse alkynes (aryl, heteroaryl, and aliphatic, including halides, esters, and boronates) and alkyl sources (primary/secondary pyridinium salts, including heterocycles, such as thiophene and indole), with gram‐scale synthesis and one‐pot, amine‐to‐product conversion. Late‐stage derivatization is used for drugs (e.g., alogliptin, vitamin E, erlotinib) to enable bioconjugation or to serve as Raman imaging tags. Radical involvement is evident from TEMPO trapping and cyclopropylmethyl ring‐opening; a Ni bis(acetylide) intermediate forms via double transmetalation, followed by oxidative addition to the pyridinium and reductive elimination. Ni(0) is unlikely, and the pincer enhances alkyl–alkynyl coupling selectivity.

**SCHEME 9:**
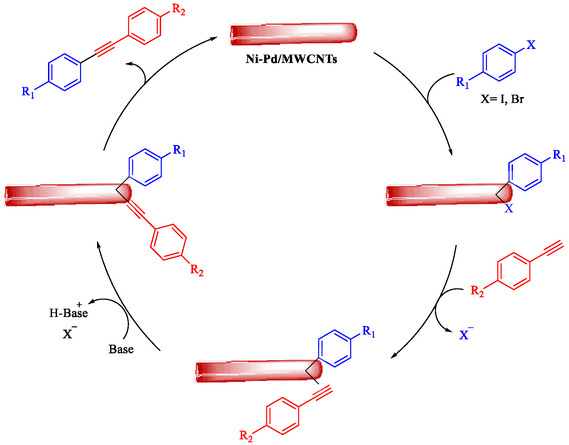
The feasible pathway for the Sonogashira coupling.

Wilson et al. developed Ni‐Pd bimetallic nanoparticles (NPs) supported on multi‐walled carbon nanotubes (Ni‐Pd/MWCNTs) *via* a solventless ball‐milling method, enabling ligand‐ and copper‐free Sonogashira reaction of Ar‐X with terminal alkynes. Optimal catalysts (e.g., 7.9 wt% Ni/0.81 wt% Pd) feature 5–10 nm particles with high dispersion (TEM), metallic Pd/Ni phases alongside NiO (XRD/XPS), and synergistic Ni–Pd electron transfer enhancing activity over monometallic analogs [69]. Reactions in H_2_O/EtOH with K_2_CO_3_ at 120°C (Monowave reactor) achieve 72%–100% conversions in 15–30 min, even at 0.01 mol% loading (turnover number (TON) 7200, TOF 21, 600 h^−1^, Table 4). The catalyst tolerates electron‐withdrawing (aldehyde, nitro, and nitrile) and electron‐donating (methoxy) groups on aryl iodides/bromides, as well as heterocycles (pyrazine, pyridine, pyrimidine) and diverse alkynes (alkyl, methoxy, amino, and propargyl alcohol), yielding 80%–99% isolated yields. Bromoarenes succeed at higher temperature/time, aligning with your Pd‐based Suzuki interests but Pd‐minimized here. Centrifugation enables 5–6 recycles (85%–100% conversion) with minimal Pd leaching, as evidenced by sustained activity. Mechanism involves oxidative addition to Ni–Pd sites, alkyne π‐coordination/deprotonation, and reductive elimination. This green, scalable approach reduces noble‐metal use in C–C couplings, complementing magnetic nanocatalysts, such as Nasseri's CuNi system. Scheme 9 represented the putative mechanism for the Sonogashira coupling by Ni–Pd/MWCNTs.

**TABLE 4:** The Sonogashira reactions using Ni‐Pd/MWCNTs.

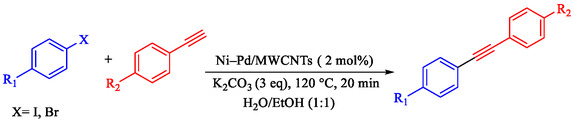				
Entry	Ar–X	Alkyne	Product	Yield, %
1	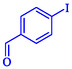	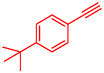	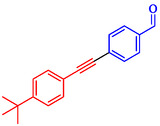	95
2	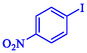	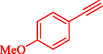	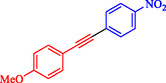	92
3	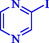	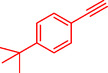	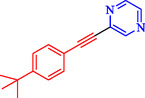	95
4	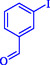	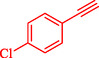	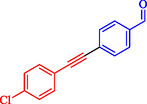	90
5	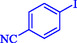	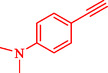	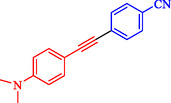	88
6	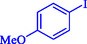	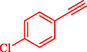	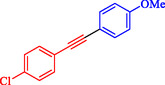	90
7	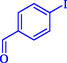	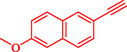	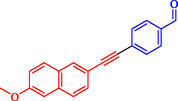	85
8	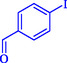	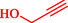	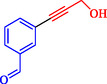	89
9	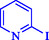	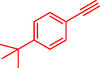	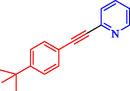	96

*Note:* Reaction condition: Ar–X (0.25 mmol), alkyne (0.3 mmol), K_2_CO_3_ (0.75 mmol), catalyst (7.50 mg, 2 mol%), and H_2_O/EtOH (1:1) (4 mL), 120°C, 15 min, Monowave 50 heating reactor.

**SCHEME 10:**
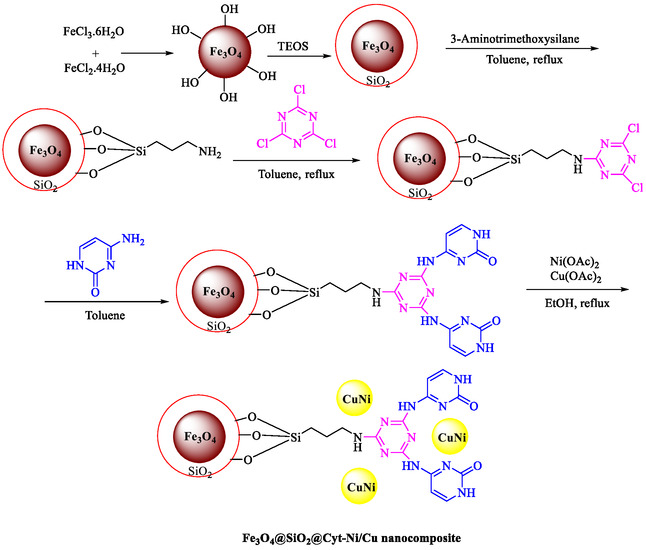
The preparation path for Fe_3_O_4_@SiO_2_@Cyt‐Ni/Cu.

Nasseri et al. developed a magnetically recyclable Fe_3_O_4_@SiO_2_‐Cyt‐NiCu bimetallic nanocatalyst for Pd‐free Sonogashira and C–N coupling reactions, achieving high yields under moderate conditions [70]. The catalyst synthesis involves sequential functionalization of silica‐coated magnetite nanoparticles with 3‐aminopropyltriethoxysilane (APTES), cyanuric chloride, cytosine, and Ni/Cu acetates, confirmed by FT‐IR, PXRD, TEM (21–24 nm particles), EDX/ICP (0.6 wt% Cu, 0.63 wt% Ni), TGA (27% organic loss), VSM (19.3 emu/g), and XPS. Scheme 10 indicates the preparation approach for Fe_3_O_4_@SiO_2_@Cyt‐Ni/Cu. DFT calculations at B3LYP/6‐31G‐LANL2DZ identified the most stable Ni/Cu binding mode on cytosine, supporting synergistic effects that enhance activity over monometallic analogs. Optimized Sonogashira conditions (10 mg catalyst, 0.1 mol% Ni/Cu, K_2_CO_3_, DMSO, 90°C, 2 h) gave 93% yield for 4‐iodotoluene/phenylacetylene and were extended to diverse aryl iodides/bromides (75%–97% yields, 1–8 h) (Table 5). C–N couplings (NaOH, water, 70°C, 2 h) with Ar–X/phenylboronic acid and *N*‐heterocycles yielded 78%–90%. Control experiments and comparisons highlight bimetallic synergy, with superior efficiency (low catalyst loading, short reaction times) compared to prior Ni/Cu systems. A dual Ni/Cu cycle mechanism involves oxidative addition (Ni), acetylide formation/transmetalation (Cu), and reductive elimination, which is similar for C–N *via* amine–Cu intermediate. The catalyst recycles seven times (87%–93% yields for Sonogashira) with unchanged structure (FT‐IR, SEM/TEM, PXRD) and minimal leaching (hot filtration/Hg‐poisoning tests). This aligns with green chemistry principles for sustainable nanocatalysis in your nitroarene/Suzuki research focus. Table 6 illustrates a comparison of the Fe_3_O_4_@SiO_2_@Cyt‐Ni/Cu catalyst with other catalysts formerly reported for the Sonogashira reaction.

**TABLE 5:** The Sonogashira reactions utilizing Fe_3_O_4_@SiO_2_@Cyt‐Ni/Cu.

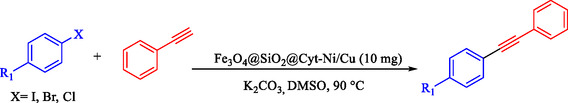				
Entry	**Ar**–**X**	Product	Time, h	Yield, %
1	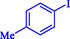	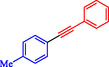	2	93
2	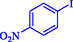	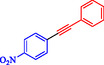	1	96
3	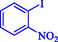	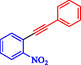	1.5	90
4	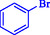	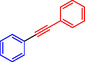	2	83
5	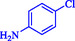	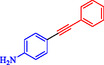	3.5	80
6	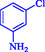	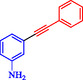	4	75
7	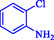	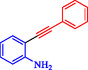	4	72
8	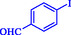	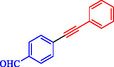	1.5	83
9	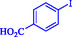	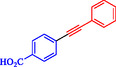	1.5	80
10	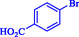	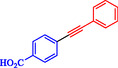	2.5	75
11	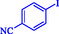	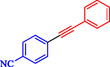	5	97
12	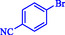	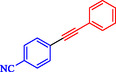	2.5	95

*Note:* Reaction conditions: Ar–X (1 mmol), phenyl acetylene (1.2 mmol), K_2_CO_3_ (2 mmol), and DMSO (3 mL), 90°C.

**TABLE 6:** A comparison of the Fe_3_O_4_@SiO_2_@Cyt‐Ni/Cu catalyst and earlier catalysts utilized in the Sonogashira coupling between phenylacetylene and 4‐iodotoluene.

Entry	Catalyst	Reaction condition	Yield, %	Ref.
1	**Fe**_**3**_ **O**_**4**_ **@SiO**_**2**_ **@Cyt‐Ni/Cu (0.1 mol%)**	**DMSO, K**_**2**_ **CO**_**3**_ **, 2 h, 90°C**	**93**	[70]
2	PdCu@GQD@Fe_3_O_4_ (Pd 0.3 mol%, Cu 0.35 mol%)	Toluene, DABCO, 24 h, 60°C	99	[71]
3	MNPs@Cs‐MS‐Co (0.55 mol%)	DMSO, KOH 10 h, 140°C	72	[72]
4	γ‐Fe_2_O_3_@Cu(II)IL‐SB (0.4 mol%)	DMSO, K_2_CO_3_, 4 h, 90°C	93	[73]

**SCHEME 11:**
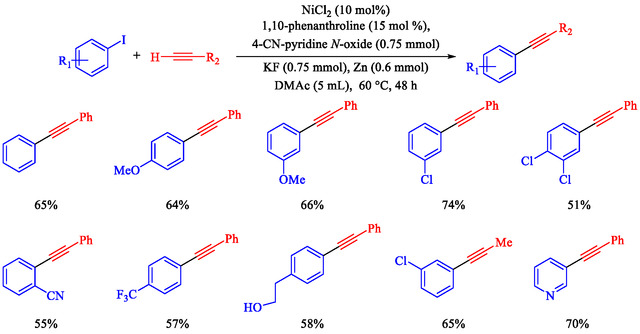
The Sonogashira coupling of terminal alkynes and Ar–I.

**SCHEME 12:**
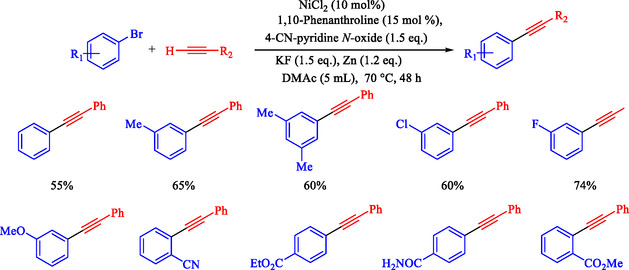
The Sonogashira coupling of terminal alkynes and Ar–Br.

Chen et al. reported that a Ni‐catalyzed Sonogashira reaction enables efficient Csp^2^—Csp bond formation between terminal alkynes and aryl iodides or bromides under mild conditions (60°C–70°C in DMAc), using NiCl_2_ (10 mol%), 1,10‐phenanthroline ligand, 4‐cyanopyridine *N*‐oxide base, KF additive, and Zn reductant; no cocatalysts are required [74]. This earth‐abundant metal system presents an expansive substrate range with high functional group tolerance (e.g., Cl, OMe, CF_3_, OH, CN, and esters). It applies to heteroaryl/gram‐scale syntheses for optoelectronics, photodynamic therapy precursors, and bioactive building blocks. Optimal conditions identified: DMAc solvent, 60°C for iodides/70°C for bromides (Schemes 11 and 12), 48 h under N_2_; alternatives like THF/toluene fail; strong bases (KO^t^Bu) cause alkyne dimerization. Yields drop without Ni, ligand, base, or Zn, confirming their necessity. Aryl iodides/bromides with electron‐neutral/donating/withdrawing groups couple easily (mild‐good yields); heteroaryls and protected alkynes are tolerated.

**SCHEME 13:**
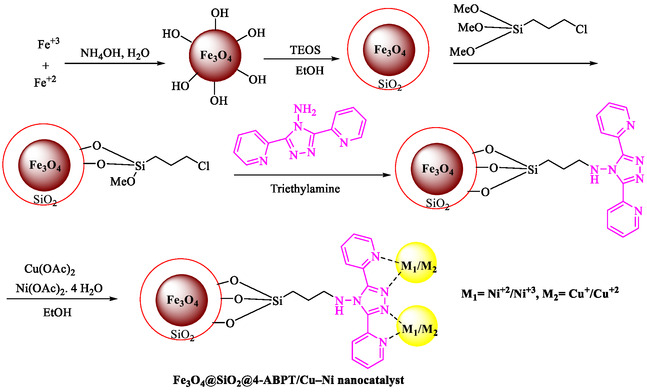
The preparation path for Fe_3_O_4_@SiO_2_@4‐ABPT/Cu–Ni.

Nasseri et al. reported the Fe_3_O_4_@SiO_2_@4‐ABPTCuNi, a magnetically recoverable Cu–Ni bimetallic nanocatalyst (0.06 mol% Ni, 0.08 mol% Cu) with a conjugated 4‐amino‐3,5‐bis(pyridin‐2‐yl)‐1,2,4‐triazole (4‐ABPT) bridge, enabling efficient Pd‐ and solvent‐free Sonogashira and C–N coupling reactions at 120°C [75]. Scheme 13 demonstrates the preparation approach for Fe_3_O_4_@SiO_2_@4‐ABPT/Cu‐Ni. The synergistic electron transfer between copper and nickel, promoted by the π‐conjugated ligand, generates active Cu(I)/Ni(III) species that outperform monometallic analogs. Fe_3_O_4_ nanoparticles are coated with SiO_2_ via Tetraethyl orthosilicate (TEOS), functionalized with chloropropyltrimethoxysilane, then anchored with 4‐ABPT and impregnated with Cu(OAc)_2_/Ni(OAc)_2_ in ethanol. Characterization (FT‐IR, XRD, TEM, XPS, VSM) confirms core‐shell morphology (∼14 nm), superparamagnetism (Ms = 43.8 emu/g), and mixed Cu(I/II)/Ni(II/III) states with uniform elemental distribution. The catalyst couples Ar‐X (I, Br, Cl) with phenylacetylene (70%–95% yields, 1–4.5 h) or *N*‐heterocycles with phenylboronic acid/ Ar–X (67%–88% yields), favoring electron‐withdrawing substituents (Table 7). Control experiments confirm bimetallic synergy (95% vs. 65%/35% for Cu/Ni mono‐systems); LSV/XPS reveal enhanced redox *via* Ni → Cu electron transfer. Recycles five times (95 → 87% yield) with minimal leaching (ICP: Cu 0.08 → 0.073 mmol/g), retaining structure/morphology. Table 8 shows a comparison of the Fe_3_O_4_@SiO_2_@4‐ABPT/Cu‐Ni NPs catalyst with some reported catalysts employed in the Sonogashira coupling between phenylacetylene and iodobenzene.

**TABLE 7:** The Sonogashira reaction using the Fe_3_O_4_@SiO_2_@4‐ABPT/Cu‐Ni catalyst.

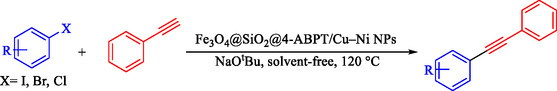				
**Entry**	**R**	**X**	**Time, h**	**Yield, %**
1	H	I	1	95
2	4‐Me	I	1.5	76
3	2‐Me	I	2.5	73
4	4‐OMe	I	3	75
5	4‐CO_2_H	I	1.5	87
6	4‐NO_2_	I	1	82
7	2‐NO_2_	I	1.5	80
8	4‐COH	I	2	90
9	H	Br	1.5	90
10	4‐NH_2_	Br	4.5	74
11	4‐CO_2_H	Br	3	76
12	4‐CN	Br	3.5	85
13	H	Cl	2.5	95
14	2‐NH_2_	Cl	4.5	75

*Note:* Reaction conditions: Ar–X (1 mmol), phenyl acetylene (1.5 mmol), NaO^t^Bu (1.0 mmol), catalyst (10 mg, 0.06 mol% Ni, 0.08 mol% Cu), solvent‐free, 120°C.

**TABLE 8:** A comparison of the Fe_3_O_4_@SiO_2_@4‐ABPT/Cu–Ni catalyst and earlier reported catalysts utilized for the Sonogashira coupling between phenylacetylene and iodobenzene.

Entry	Catalyst	Time, h	Condition	Yield, %	Ref.
1	Fe_3_O_4_@PEG/Cu–Co (0.13 mol% Co, 0.37 mol% Cu)	3.2	H_2_O/80°C/base‐free	95	[76]
2	Cu/Pd@Mod‐PANI‐3OHe (0.094 mol% Pd)	8	H_2_O/80°C/Et_3_N	90	[77]
3	Pd–Cu–PA (0.1 mol% Pd)	5	NMP/100°C/Bu_3_N	88	[78]
4	Pd/Cu@MCC‐PAMAM‐PEId (0.65 mol% Pd, 2.55 mol% Cu)	4	DMSO/80°C/K_2_CO_3_/N_2_ atmosphere	96	[79]
5	PdCu@GQD@Fe_3_O_4_ (Pd 0.3 mol%, Cu 0.35 mol%)	24	DABCO/Toluene/50°C	99	[71]
**6**	**Fe_3_O_4_@SiO_2_@4‐ABPT/Cu–Ni (0.06 mol% Ni, 0.08 mol% Cu)**	**1**	**Solvent‐free/120°C/NaO^t^Bu**	**95**	**[** **75** **]**

**SCHEME 14:**
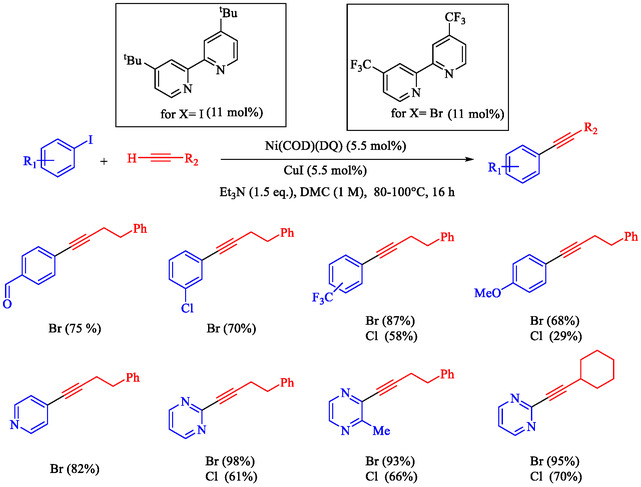
The Sonogashira reactions using the nickel catalyst.

Braconi et al. reported a Ni‐catalyzed Sonogashira reaction protocol that extends to challenging Ar–Br and Ar–Cl substrates, using air‐stable Ni(COD)(DQ), a CuI cocatalyst, and tuned bipyridine ligands in green dimethyl carbonate (DMC) solvent [80]. High‐throughput experimentation (HTE) and design‐of‐experiments (DoE) optimization revealed electron‐poor 4,4^′^‐bis(trifluoromethyl)bipyridine for bromides (80°C, Et_3_N, 16 h, 80% yield model) and electron‐rich 4,4^′^‐di‐tert‐butylbipyridine for chlorides (100°C, 52% yield model), enabling chemoselective Csp^2^–Br/Cl activation over competing halides (Scheme 14). Aryl bromides/chlorides with aldehydes, ketones, amides, cyano, esters, CF_3_, and heteroaromatics (pyridine, pyrimidine, pyrazine, thiazole) couple with alkyl/aryl alkynes in 68%–8% yields; electron‐rich substrates are slower but viable. Alkynes tolerate cyclohexyl, adamantyl, cyclopropyl, protected amines, and free alcohols; gram‐scale (73% yield) and pharmaceutically relevant products like Tazarotene (94%), MPEP (56%), and intermediates succeed, rivaling Pd methods. Ligand electronics control reactivity, with electron‐poor ligands for Br (oxidative addition), and electron‐rich ligands for Cl (nucleophilic substitution). According to Hadt's Ni‐bipy studies; no ligands like NaI are needed, mild conditions avoid DMF. This base‐metal advance reduces reliance on Pd for sustainable Csp–Csp^2^ synthesis, complementing nanoparticle systems (Nasseri CuNi, Wilson NiPd) in our green catalysis portfolio.

**SCHEME 15:**
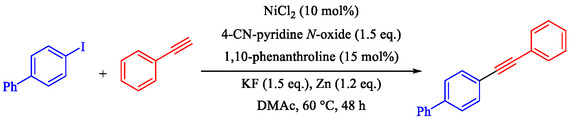
Ni‐catalyzed Sonogashira reaction of 4‐iodotoluene and phenyl acetylene.

Chen et al. presented a detailed method for Ni–catalyzed Sonogashira reactions between terminal alkynes and Ar–I or Ar–Br, thereby forming Csp^2^—Csp bonds and avoiding traditional palladium‐copper systems [81]. The authors detail the preparation of reagents, assembly of reactions, purification processes, and characterization methods, with examples yielding products like 4‐phenylethynyl‐1,1^′^‐biphenyl (65% yield, Scheme 15) and 6‐phenylethynyl‐1 phenylsulfonyl‐1*H*‐indole (74% yield). The technique uses NiCl_2_ with the 1,10‐phenanthroline ligand, 4‐cyanopyridine *N*‐oxide as the oxidant, KF as the base, and activated zinc powder in degassed DMAc under N_2_ at 60°C–70°C for 48 h. It demonstrates good functional‐group tolerance with weak bases and enables selective alkynylation of multi‐halogenated arenes. This method is restricted to aryl iodides/bromides; aryl chlorides require 120°C with a low yield (5%), and triflates give ∼20% yield. Air‐ and moisture‐sensitive steps require glovebox or Schlenk techniques.

Binandeh described the preparation and application of a novel magnetic nanocatalyst (Fe_3_O_4_@SiO_2_@CPTES@C_11_H_12_N_4_@Ni) for one‐pot Sonogashira coupling and alcohol oxidation reactions under green conditions [82]. This core‐shell nanocatalyst, prepared *via* chemical coprecipitation, features magnetite nanoparticles coated with silica and organic linkers (3‐chloropropyltriethoxysilane and 2,4‐diaminopyridine), as well as nickel metal, enabling high efficiency, recyclability, and easy magnetic separation. The nanocatalyst's structure was confirmed through multiple techniques, including SEM/TEM (particle size 80–90 nm), FT‐IR (functional group bonds like Fe—O at 588 cm^−1^ and Si—O—Si at ∼1080 cm^−1^), EDX (elemental composition), VSM (magnetization 21 emu/g), XRD (crystalline Fe_3_O_4_ peaks), TGA (thermal stability up to 800°C), XPS, BET (surface area 51 m^2^/g), and UV analyses. These confirm a stable, superparamagnetic structure with Ni nanoparticles (∼4–6 nm), thereby enhancing catalytic sites. The catalyst (0.02 mol%) facilitates double Sonogashira Csp–Csp^2^ coupling of Ar–X with phenylacetylene in DMF/K_2_CO_3_/Et_3_N at 70°C, yielding > 97% products (di/tri/quadruple triple bonds) in 15–60 min. Optimization tables show superiority over prior Pd/Ni catalysts (e.g., 99% yield vs. 80%–94% in 1–24 h, Table 9), with TON/turnover frequency (TOF) up to 73/36.5 h^−1^; products verified by NMR, IR (C≡C at ∼2200 cm^−1^), and high‐resolution mass spectrometry (HRMS). Reactions use minimal catalyst and benign solvents/temperatures and enable > 10 reuses with < 4% efficiency loss; leaching tests confirm heterogeneity. The system outperforms literature catalysts in yield, time, and sustainability, producing novel multitriple‐bond compounds for pharmaceutical applications.

**TABLE 9:** A comparison of the FeSi/CL/Ni nanocatalyst and earlier catalysts utilized in the Sonogashira reaction.

Entry	Catalyst	Time, h	Condition	Yield, %	Ref.
1	Xantphos/Pd	24	Reflux	80	[83]
2	Fe_3_O_4_/PANI/Au	12	Dioxane/90°C	92	[84]
3	Pd–PiNe	6	M.W./100°C	94	[85]
4	Pd/g‐C_3_N_4_	1	100°C	81	[86]
5	MCC‐PAMAMG2.5‐PEI/Pd/Cu (2 mol%)	30 min	DMSO/1000°C	98	[79]
6	Pd/chitin	10	CH_2_Cl_2_/90°C	90	[87]
**7**	**FeSi/CL/Ni (0.02 mol%)**	**25–60 min**	**DMF/700°C**	**99**	**[** **82** **]**

**SCHEME 16:**
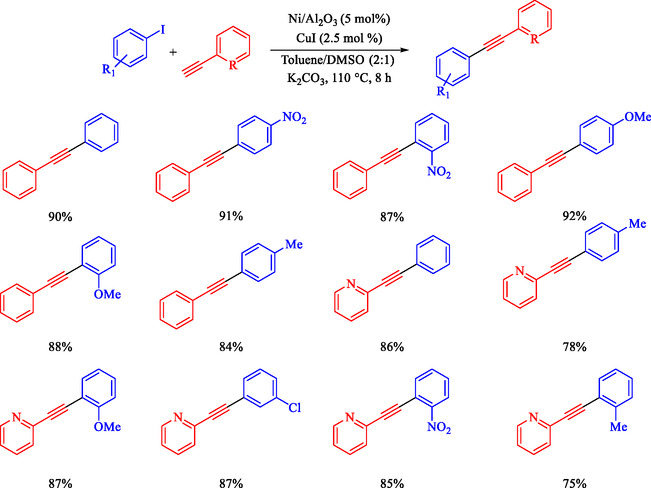
Ni‐catalyzed Sonogashira reaction of phenylacetylene with Ar–I.

Saroj and coworkers reported the development of Ni/Al_2_O_3_ as a sustainable, Pd‐free heterogeneous catalyst in the Sonogashira reaction of Ar‐I and terminal alkynes, such as phenylacetylene and 2‐ethynylpyridine [88]. Prepared *via* simple coprecipitation and characterized by SEM, EDX, and XRD, the catalyst exhibits high surface area, stability, and recyclability over six cycles with minimal Ni leaching. Optimized conditions (5 mol% Ni/Al_2_O_3_, CuI (cocatalyst), K_2_CO_3_ (base), toluene/DMSO or DMSO (solvent, 110 or 130°C) deliver 84%–92% yields across electron‐donating/withdrawing substituents (Scheme 16), outperforming prior Ni or Pd systems in cost, scalability, and green metrics. Ni/Al_2_O_3_ forms a gray powder from NiCl_2_ · 6H_2_O and alumina reduced by NaBH_4_, exhibiting uniform Ni dispersion (3.1 wt% fresh, 2 wt% after recycling), increased surface roughness post‐use, and preserved crystallinity per XRD. Hot filtration confirms heterogeneity, with no Pd toxicity or complex synthesis needed. Catalyst screening favors Ni/Al_2_O_3_ (85% yield) over NiCl_2_ or oxides, with loading optima at 5 mol%. Broad scope tolerates NO_2_, OMe, Me, Cl, and heterocycles, with para‐substituents outperforming ortho‐substituents due to steric effects. Minor Glaser byproduct observed via GC‐MS. Plausible cycle involves Ni oxidative addition to ArI, Cu‐acetylide transmetalation, and reductive elimination.

**SCHEME 17:**
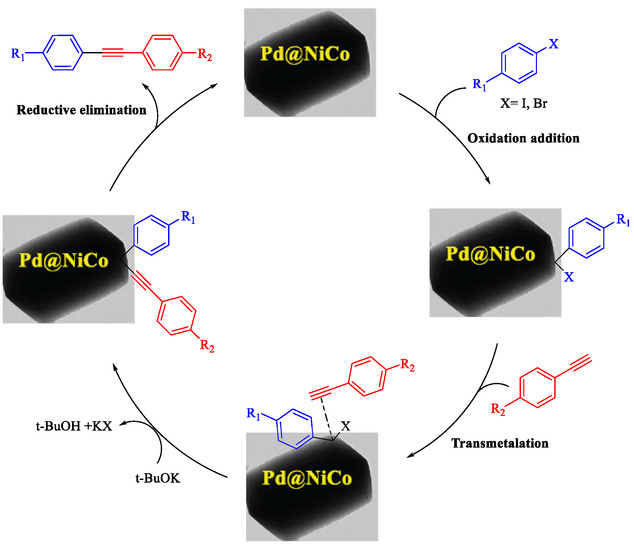
The presented a mechanism for the Pd@NiCo nanoprism‐catalyzed Sonogashira reaction.

Gholinejad et al. reported a trimetallic PdNiCo nanoprism catalyst containing ppm levels of palladium (0.06 mmol/g), synthesized via a simple reflux method in ethanol with PVP as a stabilizer, for the Sonogashira reaction between aryl/heteroaryl halides and terminal alkynes [89]. This catalyst enables efficient C—C bond formation under moderate conditions (DMF, t‐BuOK base, 60°C), achieving 91%–99% yields for diverse substrates including electron‐rich/poor iodides, bromides, and alkynes, with high turnover numbers (TON up to 7388, Table 10). PdNiCo nanoprisms form from nickel and cobalt acetates with PdCl_2_ in ethanol at 90°C for 16 h, yielding uniform prism morphologies confirmed by SEM/TEM (200–300 nm edges), elemental mapping (homogeneous Ni/Co/Pd distribution), EDX, XRD (crystalline NiCo with Pd^0^ peaks), and XPS (mixed Ni^2+^/Ni^3+^, Co^2+^/Co^3+^, Pd^0^/Pd^2+^ states). Optimization showed 9.5 ppm Pd (3 mg catalyst) suffices for 94% yield in the model iodobenzene‐phenylacetylene reaction after 10 h, outperforming monometallic/bimetallic analogs due to synergistic metal effects. It excels with challenging bromides and heteroaryl halides, surpassing literature Pd‐ppm catalysts in activity and loading. The heterogeneous catalyst recycles up to ten times with minimal Pd leaching (4–38% over cycles), retaining prism structure (slight elongation post‐use) as verified by TEM, XPS, EDX, and hot filtration/PVPy poisoning tests, confirming true heterogeneity. Table 11 indicates the comparison of the catalytic activity of Pd@NiCo and Pd‐ppm level catalysts in the Sonogashira coupling. Scheme 17 demonstrates the suggested mechanism for the Pd@NiCo nanoprism‐catalyzed Sonogashira reaction.

**TABLE 10:** Sonogashira coupling utilizing the Prism Pd@NiCo catalyst.

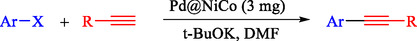							
Entry	Ar‐X	R	Time, h	T, °C	Product	Yield, %	TON
1	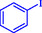	C_6_H_5_‐	10	60	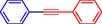	94	7015
2	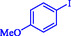	C_6_H_5_‐	10	60	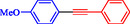	93	6940
3	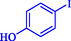	C_6_H_5_‐	10	60	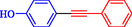	93	6940
4	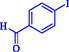	C_6_H_5_‐	10	60	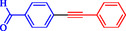	>99	7388
5	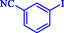	C_6_H_5_‐	10	60	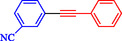	>99	7388
6	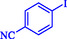	C_6_H_5_‐	10	60	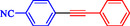	99	7388
7	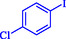	C_6_H_5_‐	10	60	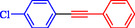	>99	7388
8	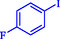	C_6_H_5_‐	10	60	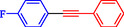	99	7388
9	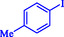	C_6_H_5_‐	10	60	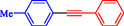	92	6866
10	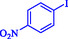	C_6_H_5_‐	101	60	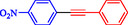	94	7015

*Note:* Reaction conditions: Ar–X (0.5 mmol), terminal alkyne (0.75 mmol), t‐BuOK (0.75 mmol), Pd@NiCo (3 mg), and DMF (2 mL).

**TABLE 11:** The comparison of the catalytic activity of Pd@NiCo and Pd‐ppm level catalysts in the Sonogashira reaction of iodobenzene with phenylacetylene.

Entry	Catalyst	Pd, ppm	Condition	Yield, %	Ref.
1	SBA‐16 supported Pd complex	200	Piperidine, 80°C, 2 h	94	[90]
2	CuI‐(PPh_3_)_2_ PdCl_2_	167	Et_2_NH, r.t. 3 h	90	[91, 92]
3	Fe/ppm pd NPs	500	H_2_O, Et_3_N, 45°C, 24 h	95	[93]
4	PdCl_2_	10	EtOH, K_2_CO_3_, 90°C, 48 h	64	[94]
**5**	**Nanoprism Pd@NiCo**	**9.5**	**DMF, t‐BuOK, 60°C, 10 h**	**94**	**[** **89** **]**

## 3 Conclusion

In summary, nickel catalysts have revolutionized Sonogashira reactions by providing earth‐abundant, less toxic alternatives to palladium, enabling copper/ligand‐free couplings of aryl/alkyl halides and terminal alkynes under milder conditions. Advances in pincer ligands, bimetallic nanoparticles, and photoredox strategies have expanded substrate scope, enhanced recyclability, and minimized side reactions such as β–H elimination. In summary, this review highlights recent advances in nickel‐catalyzed Sonogashira reactions involving diverse aromatic halides and alkynes, drawing on key literature references spanning 2012–2025. Advances in both homogeneous and heterogeneous nickel‐catalyzed Sonogashira reactions lay a robust foundation for ongoing research. Nickel‐mediated Sonogashira couplings hold considerable promise for continued expansion of substrate scope. Future research should prioritize developing copper‐ and ligand‐free nickel systems for nonactivated aryl chlorides and alkyl electrophiles, mitigating β‐hydride elimination and alkyne oligomerization through photoredox or electrocatalytic activation. Heterogeneous magnetic nanocatalysts, such as Ni‐supported on silica or graphene composites, offer promise for scalable, recyclable processes with minimal leaching, extending to enantioselective variants and continuous‐flow setups. Integrating nickel catalysis with biopolymer supports (e.g., chitosan, cellulose) could further enhance sustainability, aligning with broader goals in eco‐friendly C—C bond formation for pharmaceuticals and materials.

## 4 Outlook

Recent advances in nickel catalysts for Sonogashira reactions highlight nickel's role as a cost‐effective, abundant alternative to palladium, enabling efficient Csp–Csp^2^ and Csp–Csp^3^ couplings under milder conditions. These developments include homogeneous Ni complexes, ligand‐free systems, and heterogeneous supports like nanoparticles, with ongoing progress in alkyl electrophiles and sustainability. Nickel catalysts in Sonogashira reactions face key challenges, including poor reactivity with less‐activated aryl bromides/chlorides, β–hydride elimination with alkyl electrophiles, alkyne oligomerization, and reliance on copper cocatalysts, which complicate workups and limit substrate scope. These issues stem from sluggish oxidative addition, unstable alkyl‐Ni intermediates, and low transmetalation efficiency with sp‐hybridized alkynes. Oxidative addition of nonactivated alkyl or aryl chloride electrophiles to Ni(0) remains reluctant due to high activation barriers, often requiring harsh conditions (e.g., 140°C). β–H elimination from alkyl–Ni intermediates competes with transmetalation, reducing yields for primary/secondary alkyl substrates. Terminal alkyne side reactions, like Glaser homocoupling or cyclotrimerization, persist without precise ligand control or copper‐free designs. Future directions in nickel catalysts for Sonogashira reactions emphasize expanding substrate scope to nonactivated alkyl chlorides, achieving enantioselective couplings, and developing sustainable, copper/ligand‐free heterogeneous systems with minimal noble‐metal use. Integration of photoredox or electrocatalysis promises milder conditions and better selectivity against side reactions, such as β–H elimination. These advances align with green chemistry by prioritizing recyclable supports and earth‐abundant Ni over Pd.

## Author Contributions

**Sara Payamifar**: investigation, writing – original draft, software, conceptualization. **Pegah Etminan**: investigation, writing – original draft, visualization. **Ahmad Poursattar Marjani**: investigation, supervision, writing – review and edition.

## Conflicts of Interest

The authors declare no conflicts of interest.

## Data Availability

The data that support the findings of this study are available from the corresponding author upon reasonable request.
